# Poster Session I - A104 INSIGHTS ON A NURSE NAVIGATOR INTERVENTION FOR INDIVIDUALS WITH INFLAMMATORY BOWEL DISEASE LIVING IN RURAL COMMUNITIES: A STUDY FROM SASKATCHEWAN, CANADA

**DOI:** 10.1093/jcag/gwaf042.104

**Published:** 2026-02-13

**Authors:** N Adhikari, N Rohatinsky, S Fowler, I Boyd, J Jones, R Sanderson, U Teucher, J Peña-Sánchez

**Affiliations:** University of Saskatchewan College of Medicine, Saskatoon, SK, Canada; University of Saskatchewan College of Nursing, Saskatoon, SK, Canada; University of Saskatchewan College of Medicine, Saskatoon, SK, Canada; Kinistino, Kinistino, SK, Canada; Dalhousie University Faculty of Medicine, Halifax, NS, Canada; James Smith Cree Nation, James Smith Cree Nation, SK, Canada; University of Saskatchewan College of Arts and Science, Saskatoon, SK, Canada; University of Saskatchewan College of Medicine, Saskatoon, SK, Canada

## Abstract

**Background:**

In previous studies, we identified that rural Saskatchewan (SK) residents with inflammatory bowel disease (IBD) had fewer visits with a gastroenterologist, were less likely to have a gastroenterologist as the primary IBD care provider, had lower endoscopy rates, and had higher rates of IBD hospitalizations in comparison to persons with IBD living in urban SK. Narratives of persons with IBD residing in rural SK highlighted encountering negative experiences and health outcomes when receiving care. This evidence highlights the need for innovative models of care to improve IBD care access.

**Aims:**

In this study, we piloted a nurse navigator intervention for individuals with IBD in rural SK delivered via telephone and explored the experiences of individuals living with IBD exposed to this intervention.

**Methods:**

This qualitative study is part of a mixed-methods evaluation of a piloted nurse navigator intervention for persons with IBD living in rural SK. Individuals interacting with the IBD nurse navigator were invited to individual interviews 8 months after study enrollment. Interviews focused on gaining an understanding of participants’ perspectives on the nurse navigator intervention and ways to enhance services. Interviews were completed via Zoom. A thematic analysis approach was applied, identifying common themes.

**Results:**

In total, 10 participants agreed to participate in the interviews. Three themes were identified: benefits of the nurse navigator program, challenges with the nurse navigator intervention, and strengthening the nurse navigator care delivery. The nurse navigator intervention was deemed beneficial by individuals with IBD, specifically in terms of easy access and efficient health care navigation. Participants reported improved access to the nurse navigator, which enabled timely appointment scheduling and effective symptom management. These outcomes contributed to perceived improved disease control, reduced unnecessary specialist consultations, and decreased travel requirements.

Despite these benefits, challenges such as limited support and delayed communication were identified. Some patients experienced inconsistent contact and uncertainty during care transitions. Recommendations include enhancing communication protocols by providing direct contact information for the nurse navigator and ensuring faster response times during disease flare-ups.

**Conclusions:**

The intervention demonstrates potential to improve patient outcomes and experiences through enhanced access to health service delivery. Addressing communication gaps and expanding navigator services are necessary steps to optimize care for individuals with IBD. Continued support for this program is essential to ensure its long-term effectiveness.

**Funding Agencies:**

Saskatchewan Health Research Foundation (SHRF)

